# Sex comparisons in physiological and cognitive performance during hypoxic challenge

**DOI:** 10.3389/fphys.2022.1062397

**Published:** 2022-11-23

**Authors:** Kaila A. Vento, Cammi K. Borden, Kara J. Blacker

**Affiliations:** ^1^ Naval Medical Research Unit-Dayton Wright-Patterson Air Force Base, Dayton, OH, United States; ^2^ Oak Ridge Institute for Science and Education, Oak Ridge, TN, United States

**Keywords:** sex differences, female aviators, hypoxia, unexplained physiological events, human performance

## Abstract

Within the tactical aviation community, human performance research lags in considering potential psychophysiological differences between male and female aviators due to little inclusion of females during the design and development of aircraft systems. A poor understanding of how male and female aviators differ with respect to human performance results in unknown potential sex differences on aeromedically relevant environmental stressors, perchance leading to suboptimal performance, safety, and health guidelines. For example, previous hypoxia studies have excluded female participants or lacked a sizeable sample to examine sex comparisons. As such, progress toward sensor development and improving hypoxia familiarization training are stunted due to limited knowledge of how individual differences, including sex, may or may not underlie hypoxia symptoms and performance impairment. Investigating sex differences bridges the gap between aerospace medicine and operational health, and addressing hypoxia is one of many facets yet to be studied. In the current study, we retrospectively examined N = 6 hypoxia studies with male-female participant samples (total, N = 189; male, *n* = 118; female, *n* = 71). We explored sex as a predictor of physiological response, sensory deficits, the severity of cognitive performance declines, and symptom manifestation *via* linear and binary logistic regression models. We found that the female sex predicted lower peripheral oxygen saturation and the likelihood of headache reporting in response to hypoxic challenge, yet explained little variance when combined with age and body mass index. The sensory and cognitive performance models did not converge, suggesting high intra-individual variability. Together, sex, age, and body mass index were not the most robust predictors in responses to hypoxic challenge; we cannot infer this for sensory deficits and cognitive performance within an experimentally induced hypoxic environment. The findings have implications for improving hypoxia familiarization training, monitoring sensor development, and emergency response and recovery protocols in case of a hypoxia occurrence suitable for all aircrew. We recommend continuing to elucidate the impact of sex and intrapersonal differences in hypoxia and other aeromedically relevant stressors in tactical aviation.

## Introduction

Women comprise approximately 10% of all pilots and the proportion is in the single digits within the tactical aviation community (Air Force Personnel Center, 2022; Women in Aviation Advisory Board, 2022). Though women represent a growing force essential to flight-mission success, several unique challenges faced by women act as barriers to entering the aviation community, such as meeting current anthropometric standards for the cockpit and gear fit (da Silva et al., 2018; Marintseva et al., 2022; Moczynski et al., 2020; Women in Aviation Advisory Board, 2022). For example, prospective female aviators’ typically smaller stature and weight can create challenges in securely sitting in the cockpit and wearing issued flight attire, which may disqualify them from continuing with advanced training more so than male aviators (da Silva et al., 2018; Marintseva et al., 2022; Moczynski et al., 2020; Women in Aviation Advisory Board, 2022). Additional recruitment (e.g., no visibility and limited access to aviation), training (e.g., perceptions of tokenism and hegemonic masculine culture), and retention (e.g., restricted family planning policies and lack of promotion opportunities) blockades avert women from joining or further advancing with aviation careers (Ferla and Graham, 2019; Gorlin and Bridges, 2021; Women in Aviation Advisory Board, 2022). Nonetheless, women’s representation in aviation continues to rise, recently achieving in 2020, the first Black female Navy fighter pilot flying an F-16 Fighting Falcon, and in 2022 the first female demonstration pilot for the Blue Angels Squadron (Pawlyk, 2020; Toropin, 2022).

In addition to these barriers that impede women from entering and completing the military aviation training pipeline, human performance research lags in considering potential psychophysiological differences between male and female aviators due to little inclusion of females during the design and development of aircraft systems, contributing to their difficulties in meeting initial aviation program requirements (Bustamante-Sánchez et al., 2019; Hormeño-Holgado and Clemente-Suárez, 2019; Moczynski et al., 2020; Marintseva et al., 2022; Women in Aviation Advisory Board, 2022). Thus, a poor understanding of how male and female aviators differ in aircraft human performance results in unknown potential sex differences on aeromedically-relevant environmental stressors, perchance leading to suboptimal performance, safety, and health guidelines towards servicewomen, negatively impacting their duties (Braun et al., 2015; Eichler, 2021). Most of the work cited above that has examined the aviation community has focused on gender (e.g., men, women), demonstrating that there are fewer women compared to men in the military aviation community. While this is accurate, in the current paper, we focus instead on sex (i.e., male vs. female), as our questions of interest are primarily focused on factors that are physiologically based, such as respiratory and neural measures as well as, cognitive performance.

To illustrate, the threat of hypoxia and its possible contribution to recent unexplained physiological events (UPEs) are at the forefront of human performance research and operations in tactical aviation (Elliot and Schmitt, 2019). Reduced levels of breathable oxygen harmfully impact sensory, cognitive, and motor functioning and decision-making (McMorris et al., 2017; Niedermeier et al., 2017; Bouak et al., 2018; Hormeño-Holgado and Clemente-Suárez, 2019; Blacker et al., 2021). These hypoxia-induced performance impairments are associated with aircrews’ inability to maintain a constant airspeed, altitude, and directional heading during simulated flights (Temme et al., 2010; Kowalczuk et al., 2016; Steinman et al., 2017; Bouak et al., 2018). Hypoxia risks operational errors, mission failures, loss of aircraft, injuries, and even death (Petrassi et al., 2012; Steinman et al., 2017; Bouak et al., 2018). Therefore, efforts to precisely characterize performance impairments and recognize symptoms during hypoxia to initiate emergency procedures are demanded (Petrassi et al., 2012; Kasture et al., 2021; Leinonen et al., 2021; Blacker and McHail, 2022). While these performance deficits during acute hypoxia are well-documented, progress toward sensor development and improving hypoxia familiarization training are stunted due to limited knowledge of how individual differences, including sex, may or may not underlie hypoxia symptoms and performance impairment.

Some previous hypoxia studies have excluded female participants entirely (Kowalczuk et al., 2016; Dart et al., 2017; Steinman et al., 2017; Bouak et al., 2018; Bustamante-Sánchez et al., 2019; Lucertini et al., 2020). Whereas those that did include females lacked a large enough sample size to look at sex comparisons (Blacker et al., 2021; Fehrenbacher et al., 2021; Leinonen et al., 2021). Temme et al. (2010) and Kasture et al. (2021) do not specify participants’ sex, referring to them as pilots or volunteers. However, very recently, a few studies have begun to examine the role of sex on response to hypoxia. One such study evaluated diaphragmatic fatigue between sexes during acute hypoxia compared to normoxia (Archiza et al., 2021). The study found diaphragmatic fatigue magnitude significantly increased in female (−27.6% ± 7.7%) compared to male (−23.4% ± 9.6%) participants after induced inspiratory loading under acute hypoxia (Archiza et al., 2021). Also looking at respiratory effects of hypoxia, Camacho-Cardenosa et al. (2022) found that males had an increase in minute ventilation and a steeper initial decrease in peripheral oxygen saturation (SpO_2_) compared to females during a 7 h moderate hypoxia exposure (15% O_2_). Two additional studies examined sex differences in the effects hypoxia have on cold stress (Hohenauer et al., 2022) and muscle endurance (Karayigit et al., 2022), but both studies found no difference in SpO2 between sexes during those moderate hypoxia exposures. These inconsistent findings suggest that there may be sex differences in physiological response to hypoxic challenge. Still, differences in altitude used, normobaric *versus* hypobaric hypoxia, and length of exposure make interpretation challenging. Moreover, all of these studies had modest sample sizes (range 8–15 per sex) and none were done in an aeromedical setting. As a result, sex comparisons regarding acute hypoxia, at equivalent altitudes relevant to aviation, are non-existent, and symptoms, experiences, and performances may differ, which is critical when employing timely emergency procedures, recovery from exposure, and designing in-cockpit sensor systems.

The gravity of hypoxia adversely affecting performance and the urgency to address sex disparities within aerospace medicine merits exploring male and female aviators’ responses to low-oxygen events to equip early warning systems better. Examining sex comparisons in hypoxic challenge is an initial step towards addressing the numerous aircrew occupational stressors and environmental exposures that have been ignored, along with G-tolerance, workload, spatial disorientation, inflammatory responses, and urinary relief systems (Lewkowicz et al., 2018; Lefèvre et al., 2019; Belt et al., 2020; Chiang et al., 2021; Schultz et al., 2022). Female aircrew play an integrative role in aviation, and as their occupational opportunities expand, so should the operational health sciences to best support all aircrew needs.

Investigating sex differences bridges the gap between aerospace medicine and operational health, and addressing hypoxia is one of many facets yet to be studied. Therefore, we retrospectively examined six hypoxia studies with male-female participant samples. We explored sex as a predictor of physiological response, sensory deficits, the severity of cognitive performance declines, and symptom manifestation *via* stepwise linear and binary logistic regression analyses. We hypothesized sex (i.e., male vs. female) as a predictor of response to hypoxic challenge. The alternative hypothesis was sex would not be a predictor of the response outcomes during hypoxia.

## Materials and methods

### Study design

**FIGURE 1:**
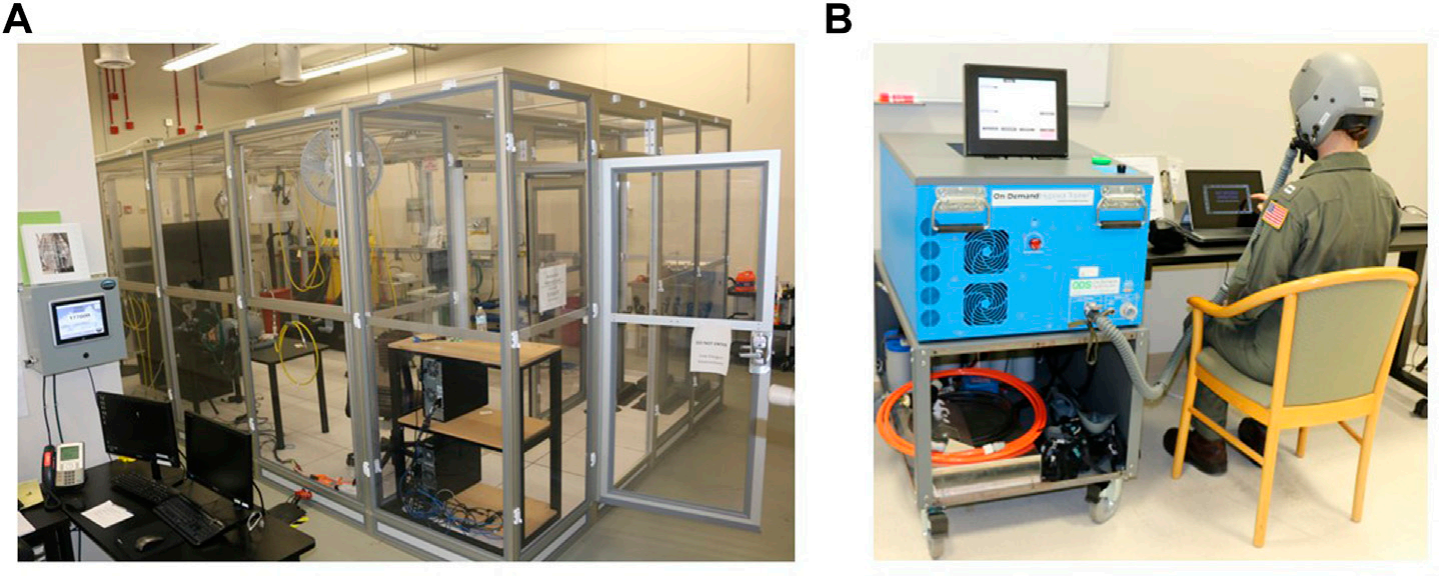
**(A)** Reduced Oxygen Breathing Environment (ROBE) where studies 1–4 and 6 took place. **(B)** On-Demand Hypoxia Trainer (ODHT) utilized for study 5. *Photos courtesy of NAMRU-D*.

A retrospective cohort study design examined N = 6 hypoxia studies, with a total of N = 189 participants datasets (male, *n* = 118; female, *n* = 71) collected from 2017 to 2022 at the Naval Medical Research Unit- Dayton (NAMRU-D); studies 1-4 published data (Seech et al., 2020; Blacker et al., 2021; Blacker and McHail, 2021; Blacker and McHail, 2022) and studies 5-6 unpublished data (completed in 2022). The purpose of these six studies was to examine physiological responses, sensory function, cognitive performance, and symptom presentation during an acute hypoxic exposure. All six studies utilized a within-subjects design that included a normobaric hypoxia exposure along with a normoxia control condition on a separate day. All had a minimum exposure time of 10 min and a minimum altitude of 10,000 ft. Variations in exposure time (10–27 min) and altitude (10,000–25,000 ft) are noted (Table 1). Five studies were conducted in a Reduced Oxygen Breathing Environment (ROBE; Hypoxico, Inc.) and one with an On-Demand Hypoxia Trainer (ODHT; Lynntech, Inc.). The ROBE is a normobaric chamber that creates simulated altitudes by delivering precise amounts of oxygen and nitrogen and does not require participants to wear a mask (Figure 1A). In contrast, the ODHT is a compact, tankless device delivering gas mixtures *via* a hose hooked up to a flight mask (Figure 1B).

**TABLE 1:** Hypoxia studies for retrospective analyses.

Study name	Sample size	Altitude (O_2_%) exposure minutes	Study outcomes
Study 1: Seech et al. (2020)	N = 40 male, n = 27, female, n = 13	17,500 ft (10.6%), 27-min	SpO_2_, HR, hypoxia-related symptoms, ERPs, VTT
Study 2: Blacker et al. (2021)	N = 29 male, n = 21		
	17,500 ft (10.6%), female, n = 8	17,500 ft (10.6%), 27-min, 27-min	SpO_2_, HR, hypoxia-related symptoms, ERPs, VTT
Study 3: Blacker & McHail (2021)	N = 31 male, n = 17, female, n = 14	20,000 ft (9.7%), 10-min	SpO_2_, HR, hypoxia-related symptoms, ERPs, PVT
Study 4: Blacker & McHail (2022)	N = 34 male, n = 16, female, n = 18	20,000 ft (9.7%), 15-min	SpO_2_, HR, hypoxia-related symptoms, ERPs
Study 5: Unpublished data^a^	N = 34 male, n = 21, female, n = 13	10,000–25,000 ft (14.3–8.1%), 20-min	SpO_2_, HR, hypoxia-related symptoms, ERPs, HAT
Study 6: Unpublished data^a^	N = 21 male, n = 16, female, n = 5	20,000 ft (9.7%), 15-min	SpO_2_, HR, hypoxia-related symptoms, ERPs, CCT

*Note*. Abbreviations (SpO_2_, peripheral oxygen saturation; HR, heart rate; ERPs, event-related potentials; VTT, visuomotor tracking task; PVT, psychomotor vigilance task; HAT, Hypoxia Awareness Tool [visuomotor, cognitive, and working memory tasks]; CCT, cone contrast task).

^a^ Studies 5 and 6 completed in 2022.

### Participants

All participants were between 18 and 40 years of age and self-reported normal or corrected-to-normal vision, normal hearing, no history of psychological, neurological, or medical diagnosis, no use of tobacco in the past 6 months, and no excessive alcohol use. Also, participants had not traveled to altitudes >5,000 ft for >10 days in the past 6 months, donated blood in the past 30 days, nor were pregnant. All participants were recruited through flyers and online announcements. All studies were approved by the NAMRU-D’s Institutional Review Board. Each participant provided written informed consent before participating. All received a gift card (s) for contributing to their respective study.

### Measurements

#### Physiological monitoring

Peripheral oxygen saturation (SpO_2_) and heart rate (HR) were monitored and recorded using a finger-mounted pulse oximeter (Nonin Connect Model 3,230, Nonin Medical, Plymouth, MN, United States ). The data were recorded and monitored using an iPad *via* Bluetooth connection. The safety cut-off value for SpO_2_ varied between 55% and 60%, depending on the altitude and length of exposure. For SpO_2_ and HR, we created absolute change variables by subtracting average normoxia values for each individual from minimum hypoxia SpO_2_ and maximum hypoxia HR, respectively. Thus, for SpO2 we expect negative values and for HR we expect positive values, due to the hypoxic ventilatory response (Lipton et al., 2010).

#### Event-related potentials

Cognitive and sensory function impairment were measured *via* ERPs (i.e., P3a, mismatch negativity [MMN], and visual P100). Electroencephalogram (EEG) data were continuously recorded from 32 or 64 electrodes using an elastic electrode cap that uniformly covered the whole scalp (Brain Products GmbH, Gilching, Germany). Electrode impedance for all channels was kept below 10 kΩ. Data were segmented into epochs 100 ms before and 400–500 ms after the stimulus presentation. Low and high-pass filters, independent component analysis (ICA), and visual inspection were conducted to reject artifacts and noise. Additional processing details can be found in each respective publication or upon request from the author for the unpublished data. The ERP average waveforms were calculated for normoxia and hypoxia exposure visits separately and then a relative change score was calculated with negative values indicating percent reductions in amplitude under hypoxic conditions compared to normoxia.

#### Cognitive performance

Of the 6 studies, 5 included continuous cognitive performance tasks, as detailed in Table 1. The four tasks used included a visuomotor tracking task (VTT), psychomotor vigilance task (PVT; Dinges and Powell, 1985), hypoxia awareness tool (HAT), and a modified cone contrast task (CCT; Rabin et al., 2011). All tasks were presented on a screen at a prescribed distance from the participant. VTT involved using a joystick, PVT and CCT involved pressing a key on a keyboard, and HAT involved pressing items on a touch screen tablet. Performance was analyzed by error for VTT, median reaction time (RT) for PVT, and accuracy for HAT and CCT (negated), which was used to calculate a relative change between the normoxia and hypoxia exposures.

#### Self-reported hypoxia-related symptoms

Participants self-reported (yes/no) hypoxia-related symptoms experienced either with a checklist recorded by a research team member or by completing the Hypoxia Symptom Questionnaire (HSQ; Sausen et al., 2001). The symptoms included tingling, hot flashes, dizziness, tunnel vision, light dimming, euphoria, loss of coordination, headache, fatigue, breathlessness, blurred vision, nausea, and apprehension. In addition, a hypoxia-related symptoms frequency score was calculated by tallying the number of reported symptoms during the hypoxia exposure.

### Statistical analyses

The Statistical Package for Social Sciences (SPSS) Version 27 was used for statistical analyses. Personal demographic, physiological, ERP, cognitive performance, and hypoxia-related symptom values are given as frequencies (*n*), percentages (%), and mean ± standard deviations (*M* ± *SD*). We conducted stepwise regression analyses, grouping Model 1 to include personal characteristics (i.e., sex, age, and BMI) and Model 2 external factors (i.e., altitude and exposure minutes) to determine how each group contributed to the variance explained. For continuous variables, separate stepwise linear regression models analyzed the independent variables (i.e., Model 1 = sex, age, and BMI; Model 2 = altitude and exposure time) on the dependent variables (i.e., SpO_2_, HR, ERP, cognitive performance, and hypoxia-related symptom frequency score). Additional binary (yes/no response) logistical regression models analyzed the above independent variables on each hypoxia-related symptom (i.e., tingling, hot flashes, dizziness, tunnel vision, light dimming, euphoria, loss of coordination, headache, fatigue, breathlessness, blurred vision, nausea, and apprehension). We included the beta (β) or odds ratio (OR), 95% confidence intervals (95% CI), and *p*-values for each dependent variable per model, along with the variance explained (*R* ^2^). All statistical analyses were performed with a significance level of *p* < 0.05.

Given the data were already collected, an *a priori* sample size calculation was not appropriate (Kim & Seo, 2013). However, we conducted a post-hoc power analysis calculating the confidence intervals around the observed effect sizes accompanied by a sensitivity analysis on the primary outcome, SpO_2_, resulting in 98% power and an effect size of 0.22.

## Results

### Participants

A total N = 189 participant datasets were combined from all 6 experimental studies. Participants frequently return to NARMU-D to partake in multiple research projects, possibly participating in more than one hypoxia study. Thus, after screening for repeated participants amongst the studies, *n* = 54 datasets were excluded. An additional *n* = 19 cases had substantial missing data, which were removed, resulting in N = 116 participant datasets for statical analyses. Of this sample, participants were male, *n* = 78 (age, 29.53 ± 5.87; height in, 70.13 ± 2.85; weight lbs., 188.13 ± 29.11; BMI, 26.82 ± 3.39) and female, n = 38 (age, 29.29 ± 5.47; height in, 64.28 ± 2.29; weight lbs., 152.89 ± 22.55; BMI, 26.03 ± 3.80). More male than female participants were included in the analysis, χ^2^ (1) = 13.79, *p* < 0.01.

### Physiological monitoring

**FIGURE 2:**
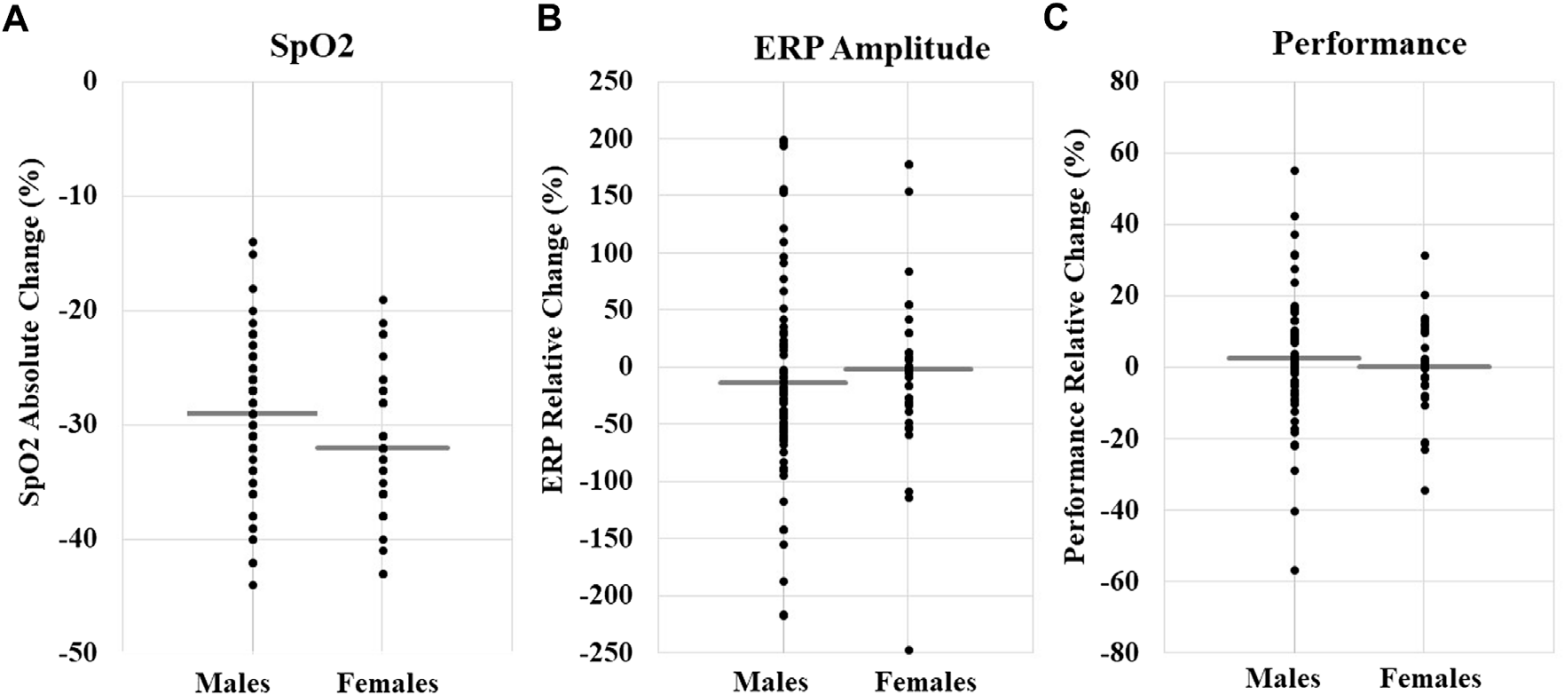
**(A)** SpO_2_ absolute change (%) by sex; total N *M*±*SD,* −30.15 ± 6.17. **(B)** ERP relative change (%) by sex; total N *M*±*SD,* −11.92 ± 87.82. **(C)** Cognitive performance relative change (%); total N *M*±*SD,* 2.03 ± 15.62. Each data point represents an individual participant’s change value, and the gray line is the mean for each sex. The figure is a visualization of data and does not represent the results of a statistical test.

For SpO_2_ and HR, we created absolute change variables by subtracting average normoxia values for each individual from minimum hypoxia SpO_2_ and maximum hypoxia HR, respectively. Those change variables were used as our dependent variables. We conducted separate stepwise linear regression analyses examining the independent variables (i.e., Model 1 = sex, age, and BMI; Model 2 = altitude and exposure time) on the dependent variables SpO_2_ and HR. Descriptive outcomes are shown in Table 2 and regression results are shown in Table 3. Models 1 (*p* = 0.07) and 2 (*p* < 0.001) variances explained for SpO_2_ were 6% and 18% (N = 115), respectively. Regarding Model 2, female sex (*p* = 0.01), altitude (*p* < 0.001), and exposure minutes (*p* = 0.038) significantly predicted decreases in SpO_2_. For HR, Models 1 (*p* = 0.02) and 2 (*p* = 0.02) variances explained were 8% and 11%, respectively. Age significantly predicted decreases in HR in Model 1 (*p* = 0.01) and remained significant in Model 2 (*p* = 0.01). No other independent variables were significantly associated with SpO_2_ and HR. Overall, personal characteristics alone explained little variance in physiological responses and were more influenced by external factors. As a visualization of the descriptive statistics in Table 3 and Figure 2A shows individual participant data for the absolute change in SpO_2_ by sex.

**TABLE 2:** Descriptive information of physiological, ERPs, cognitive performance, and self-reported hypoxia-related symptom outcomes by sex.

	Total N = 116	Male *n* = 78	Female *n* = 38
		*M*±*SD*	
SpO_2_ absolute change (%)^a^	−30.15 ± 6.17	−29.57 ± 5.99	−31.37 ± 6.47
HR absolute change (bpm)	28.53 ± 14.48	28.11 ± 14.43	29.42 ± 14.79
ERPs relative change (%)^b^	−11.92 ± 87.82	−19.38 ± 90.33	−3.02 ± 82.02
Cognitive performance relative change (%)^c^	2.03 ± 15.62	3.05 ± 16.48	−0.01 ± 13.80
Hypoxia-related symptom frequency score (*n*)	3.51 ± 2.60	3.32 ± 5.51	3.89 ± 3.04
		*n* (%)	
Hypoxia-related symptom			
Tingling	40 (35)	30 (39)	10 (26)
Hot flashes	26 (22)	16 (21)	10 (26)
Dizziness	36 (31)	20 (26)	16 (42)
Tunnel vision	41 (35)	26 (33)	15 (40)
Light dimming	26 (22)	18 (23)	8 (21)
Euphoria	13 (11)	10 (13)	3 (3)
Loss of coordination	23 (20)	15 (19)	8 (21)
Headache	24 (21)	12 (15)	12 (32)
Fatigue	60 (52)	40 (51)	20 (53)
Breathlessness	40 (35)	24 (31)	16 (42)
Blurred vision	40 (35)	26 (33)	14 (37)
Nausea	17 (15)	11 (14)	6 (16)
Apprehension	22 (19)	12 (15)	10 (26)

*Note*. Abbreviations (M±SD, mean ± standard deviation; SpO, peripheral capillary oxygen saturation; HR, heart rate; ERPs, event-related potentials). All percentages rounded to the nearest whole number. Each n (%) self-reported as “yes.”

^a^ SpO_2_ missing one participant, n = 115.

^b^ ERPs, eight participants missing, n = 108.

^c^ Cognitive performance, n = 90; 12 participants did not perform cognitive performance tasks as part of the study, 14 participants missing.

**TABLE 3:** Linear regression models of physiological and self-reported hypoxia-related symptom frequency outcomes.

	Model 1				Model 2			
	β	95% CI	*p*	*R* ^2^	β	95% CI	*p*	*R* ^2^
SpO_2_ (%)				0.06				0.18
Sex^a^	−2.67	(−5.06, −0.29)	0.03−		−3.13	−5.42, −0.83)	0.01^**^	
Age	0.14	(−0.05, −0.29)	0.15		0.14	(−0.04, 0.33)	0.13	
BMI	−0.31	(−0.45, 0.19)	0.42		−0.10	(−0.40, 0.20)	0.52	
Altitude (k)^b^					−0.86	(−1.31, −0.42)	0.01^**^	
Exposure (min)^c^					−0.18	(−0.35, −0.01)	0.04−	
HR (bpm)				0.08				0.11
Sex	1.16	(−4.08, 6.39)	0.66		1.50	(−3.80, 6.81)	0.58	
Age^d^	−0.69	(−1.12, −0.26)	0.01^**^		−0.69	(−1.12, −0.26)	0.01^**^	
BMI	0.01	(−0.69, 0.72)	0.97		−0.02	(−0.72, 0.68)	0.96	
Altitude (k)					0.18	(−0.09, 1.97)	0.07	
Exposure (min)					0.15	(−0.25, 0.55)	0.46	
Hypoxia-related symptomfrequency score (*n*)				0.01				0.22
Sex	0.58	(−0.45, 1.62)	0.27		0.61	(−0.33, 15.6)	0.20	
Age	0.02	(−0.06, 0.11)	0.59		0.03	(−0.05, 0.10)	0.51	
BMI	0.01	(−0.13, 0.15)	0.91		0.00	(−0.12, 0.13)	0.99	
Altitude (k)^e^					0.47	(0.28, 0.65)	0.01^**^	
Exposure (min)					0.01	(−0.06, 0.08)	0.73	

*Note*. Abbreviations (β = beta; CI, confidence interval; *R* ^2^ = variance explained; SpO_2_ = peripheral capillary oxygen saturation; BMI, body mass index; HR, heart rate; ERPs, event-related potentials). Male reference value for sex. SpO_2_ and heart absolute change between normoxia and hypoxia exposures.

^a^ Female sex significantly predicted decreased SpO_2,_ *p* = .01.

^b^ Altitude significantly predicted decreased SpO_2_, *p* < .001.

^c^ Exposure minutes significantly predicted decreased SpO_2_, *p* = .04.

^d^ Age significantly predicted decreased HR, *p* = .01.

^e^ Altitude significantly predicted increased hypoxia-related symptom frequency scores, *p* < .001. ^**^ *p* < .01. ^*^ *p* < .05.

### Event-related potentials and cognitive performance

Stepwise linear regression models examined the independent variables (i.e., Model 1 = sex, age, and BMI; Model 2 = altitude and exposure time) on the dependent variables ERP amplitude and cognitive performance. Models 1 and 2 for ERPs (Model 1, R^2^ = 0.02, *p* = 0.53; Model 2, R^2^ = 0.04, *p* = 0.53) and cognitive performance (Model 1, R^2^ = 0.04, *p* = 0.26; Model 2, R^2^ = 0.04, *p* = 0.51) did not have a statistically significant explained variance, therefore, the models were a poor fit (descriptive outcomes, Table 2 and Figures 2B,C). The factors predicting ERPs and cognitive performance cannot be interpreted.

### Self-reported hypoxia-related symptoms

Similarly, we conducted a stepwise linear regression analysis examining the independent variables (i.e., Model 1 = sex, age, and BMI; Model 2 = altitude and exposure time) on the dependent variable hypoxia-related symptom frequency score. Models 1 (*p* = 0.68) and 2 (*p* < 0.001) variances explained were 1% and 22%, respectively. Only altitude significantly predicted increased symptom reporting, *p* < 0.001 (descriptive outcomes, Table 2; regression outcomes, Table 3).

Binary logistic regression models examined the independent variables (i.e., Model 1 = sex, age, and BMI; Model 2 = altitude and exposure time) on the dependent variables (i.e., tingling, hot flashes, dizziness, tunnel vision, light dimming, euphoria, loss of coordination, headache, fatigue, breathlessness, blurred vision, nausea, and apprehension). Models 1 and 2 yielded the following OR and *p*-values for each symptom and associated independent variable. Female sex was significantly associated with increased reports of headache in Model 1 (OR = 2.69, *p* = 0.04) and remained significant in Model 2 (OR = 3.33, *p* = 0.02). Regarding Model 2, age was significantly associated with increased reports of hot flashes (OR = 1.12, *p* = 0.01), headache (OR = 1.05, *p* = 0.02), and fatigue (OR = 0.92, *p* = 0.02). Altitude was significantly associated with increased reports of tingling (OR = 1.37, *p* < 0.001), dizziness (OR = 1.55, *p* < 0.001), tunnel vision (OR = 1.19, *p* = 0.04), loss of coordination (OR = 1.11, *p* < 0.001), headache (OR = 1.28, *p* = 0.01), breathlessness (OR = 1.18, *p* = 0.04), and apprehension (OR = 1.59, *p* < 0.001). Exposure minutes were significantly associated with decreased reports of hot flashes (OR = 0.91, *p* = 0.03) (descriptive outcomes, Table 2; regression outcomes, Table 4).

**TABLE 4:** Binary logistic regression models of individual self-reported hypoxia-related symptoms.

	Model 1			Model 2		
	OR	95% CI	*p*	OR	95% CI	*p*
Tingling						
Sex	0.59	(0.25, 1.40)	0.24	0.64	(0.26, 1.60)	0.34
Age	1.03	(1.00, 1.10)	0.45	1.03	(0.96, 1.11)	0.40
BMI	1.05	(0.94, 1.17)	0.40	1.05	(0.93, 1.18)	0.48
Altitude (k)^a^				1.37	(1.15, 1.63)	0.01^**^
Exposure (min)				1.05	(0.98, 1.12)	0.18
Hot flashes						
Sex	1.44	(0.56, 3.71)	0.45	1.27	(0.45, 3.57)	0.65
Age^b^	1.12	(1.03, 1.21)	0.01^**^	1.12	(1.03, 1.23)	0.01^**^
BMI	0.97	(0.85, 1.11)	0.67	0.97	(0.84, 1.11)	0.65
Altitude (k)				1.21	(1.00, 1.48)	0.05
Exposure (min)^c^				0.91	(0.83, 0.99)	0.03^*^
Dizziness						
Sex	2.13	(0.93, 4.86)	0.07	2.47	(0.93, 6.57)	0.07
Age	1.02	(0.95, 1.09)	0.65	1.03	(0.95, 1.11)	0.52
BMI	1.00	(0.90, 1.13)	0.94	1.00	(0.88, 1.14)	0.99
Altitude (k)^d^				1.55	(1.27, 1.89)	0.01^**^
Exposure (min)				0.95	(0.87, 1.03)	0.22
Tunnel vision						
Sex	1.22	(0.54, 2.75)	0.64	1.22	(0.52, 2.87)	0.65
Age	1.00	(0.94, 1.07)	0.98	1.00	(0.94, 1.07)	0.97
BMI	0.91	(0.81, 1.02)	0.17	0.91	0.81, 1.02)	0.100
Altitude (k)^e^				1.19	(1.01, 1.39)	0.04^*^
Exposure (min)				1.00	(0.94, 1.07)	0.98
Light dimming						
Sex	0.85	(0.32, 2.23)	0.73	0.86	(0.32, 2.31)	0.76
Age	1.08	(1.00, 1.17)	0.05	1.08	(1.00, 1.16)	0.05
BMI	0.94	(0.82, 1.07)	0.33	0.93	(0.93, 0.86)	0.31
Altitude (k)				1.08	(0.90, 1.29)	0.40
Exposure (min)				1.00	(0.93, 1.08)	0.93
Euphoria						
Sex	0.53	(0.13, 2.08)	0.36	0.69	(0.17, 2.84)	0.61
Age	1.01	(0.91, 1.12)	0.85	1.01	(0.91, 1.12)	0.86
BMI	0.90	(0.75, 1.08)	0.25	0.88	(0.73, 1.07)	0.20
Altitude (k)				1.05	(0.82, 1.35)	0.68
Exposure (min)				1.11	(0.99, 1.25)	0.07
Loss of coordination						
Sex	1.12	(0.42, 2.94)	0.83	1.45	(0.44, 4.79)	0.54
Age	1.02	(0.95, 1.11)	0.95	1.05	(0.95, 1.15)	0.40
BMI	0.99	(0.87, 1.13)	0.87	0.96	(0.82, 1.17)	0.57
Altitude (k)^f^				1.71	(1.37, 2.12	0.01^**^
Exposure (min)				1.01	(0.91, 1.12)	0.88
Headache						
Sex^g^	2.69	(1.04, 7.00)	0.04^*^	3.33	(1.18, 9.43)	0.02^*^
Age^h^	0.90	(0.83, 0.99)	0.03^*^	0.90	(0.82, 0.99)	0.02^*^
BMI	1.03	(0.90, 1.17)	0.68	1.02	(0.90, 1.17)	0.73
Headache						
Altitude (k)^i^				1.28	(1.06, 1.54)	0.01^**^
Exposure (min)				1.05	(0.97, 1.15)	0.24
Fatigue						
Sex	1.11	(0.50, 2.48)	0.80	0.98	(0.43, 2.26)	0.96
Age^j^	0.93	(0.87, 0.99)	0.03^*^	0.92	(0.86, 0.99)	0.02^*^
BMI	0.14	(097, 1.21)	0.14	1.10	(0.98, 1.23)	0.10
Altitude (k)				0.84	(0.71, 1.00)	0.05
Exposure (min)				0.95	(0.89, 1.01)	0.10
Breathlessness						
Sex	1.78	(0.78, 4.04)	0.17	1.76	(0.75, 4.13)	0.19
Age	1.03	(0.96, 1.10)	0.50	1.03	(0.96, 1.10)	0.45
BMI	1.08	(0.97, 1.21)	0.16	1.09	(0.97, 1.22)	0.16
Altitude (k)^k^				1.18	(1.01, 1.40)	0.04^*^
Exposure (min)				0.98	(0.02, 105)	0.57
Blurred vision						
Sex	1.07	(0.47, 2.45)	0.88	1.07	(0.46, 2.50)	0.88
Age	1.00	(0.94, 1.08)	0.92	1.00	(0.94, 1.08)	0.91
BMI	0.89	(0.79, 1.01)	0.06	0.89	(0.79, 1.00)	0.06
Altitude (k)				1.05	(0.89, 1.24)	0.57
Exposure (min)				1.00	(0.94, 1.07)	0.96
Nausea						
Sex	1.26	(0.42, 3.80)	0.68	1.32	(0.43, 4.08)	0.63
Age	1.05	(0.95, 1.15)	0.35	1.05	(0.95, 1.15)	0.36
BMI	1.12	(0.97, 1.29)	0.13	1.11	(0.96, 1.29)	0.15
Altitude (k)				1.10	(0.90, 1.35)	0.37
Exposure (min)				1.00	(0.92, 1.09)	0.95
Apprehension						
Sex	2.06	(0.79, 5.39)	0.14	3.20	(0.98, 10.40)	0.05
Age	0.99	(0.91, 1.08)	0.80	0.99	(0.90, 1.09)	0.88
BMI	1.07	(0.94, 1.21)	0.34	1.07	(0.92, 1.23)	0.40
Altitude (k)^l^				1.59	(1.29, 1.60)	0.01^**^
Exposure (min)				1.02	(0.92, 1.13)	0.71

*Note*. Abbreviations (OR, odds ratio; CI, confidence interval; BMI, body mass index). Male reference value for sex.

^a^ Altitude significantly associated with increased reports of tingling, *p* < .001.

^b^ Age significantly associated with increased reports of hot flashes, *p* = .01.

^c^ Exposure minutes significantly associated with decreased reports of hot flashes, *p* = .03.

Altitude significantly associated with increased reports of dizziness, *p* < .001.

^e^ Altitude significantly associated with increased reports of tunnel vision, *p* = .04.

^f^ Altitude significantly associated with increased reports of loss of coordination, *p* < .001.

^g^ Female sex significantly associated with increased reports of headache, *p* = .02.

^h^Age significantly associated with increased reports of headache, *p* = .02.

^i^ Altitude significantly associated with increased reports of headache, *p* = .01.

^j^ Age significantly associated with increased reports of fatigue, *p* = .02.

^k^ Altitude significantly associated with increased reports of breathlessness, *p* = .04.

^l^ Altitude significantly associated with increased reports of apprehension, *p* < .001.^**^ *p* < .01.^*^ *p* < .05.

## Discussion

The current study retrospectively examined sex as a predictor of physiological response, neural modulation, cognitive performance, and hypoxia-related symptom manifestation during hypoxic challenge. Using regression analyses, we found female sex predicted lower SpO_2_ and higher headache reports in response to hypoxia. However, sex did not predict HR changes, hypoxia-related symptom frequency, or other individual symptom presentation. While sex was our primary independent variable of interest, we also examined age, BMI, altitude, and exposure minutes. Age significantly predicted decreased HR and was associated with increased reports of hot flashes, headaches, and fatigue. Surprisingly, exposure minutes were associated with a decrease in hot flashes, possibly influenced by the initial apprehension of a participant perceiving a hypoxic exposure. The results for ERPs and cognitive performance yielded poorly fitted models and, therefore, cannot be determined as the findings could be misleading when making inferences about the predictors in the model. Lastly, and expectedly, increased altitude significantly predicted lower SpO_2_, higher hypoxia-related symptom frequency scores, and increased reports of several individual symptoms documented in previous studies (Kasture et al., 2021; Blacker ad McHail, 2022).

Overall, personal characteristics had minimal bearing on physiological response (Table 3). Focusing on sex, SpO_2_ was significantly lower among female than male participants and in combination with age and BMI, explained 6% of the variance. Though accounting for a small percentage of SpO_2_, these results are consistent with Archiza et al. (2020) findings regarding sex differences in diaphragmatic muscle fatigue under acute hypoxia. However, our finding of lower SpO_2_ for females is inconsistent with prior work using less severe altitudes that found no sex differences (Hohenauer et al., 2022; Karayigit et al., 2022). The altitudes examined here most closely align with the 8% O_2_ used by Archiza et al. (2020). Moreover, given the small sample sizes used in previous work (Hohenauer et al., 2022; Karayigit et al., 2022), their null result for sex differences in SpO_2_ was possibly due to inadequate power. The larger sample size used in our study afforded us a better opportunity to assess these differences, and our results did indicate that the effect was quite small with sex, age, and BMI only accounting for 6% variance in SpO_2_ (Table 3).

Additionally, the likelihood of reporting a headache was 2.69 times more likely among female than male participants and increased to 3.33 times when adding altitude and exposure minutes (Table 4). Previous work has not specifically examined sex differences in self-reported symptoms following acute hypoxia. In general, headaches and migraines are more common among women in both military and non-military populations (Guo et al., 2019; Moore et al., 2019; Allais et al., 2020; Hesselbrock and Haynes, 2022). Increased report of headache during hypoxia for females is particularly relevant in an aeromedical context because headache frequency is a major factor influencing suitability for aviation service (Hesselbrock and Haynes, 2020). Hormonal contraceptive use is related to cerebrovascular and cognitive changes, which could be further altered during hypoxic conditions; future works should explore the association between hormonal contraceptive use and headache reports (O’Brien et al., 2020; Theunissen et al., 2022). The risk of migraine recurrence is also an important factor for aeromedical readiness, particularly in pilot applicants who incur a substantial training investment.

Here we were interested in whether males and females differ in their neurocognitive response to acute hypoxia because this is particularly relevant to military aviation. However, our ERP and cognitive performance measures yielded a poorly fitted model, which makes the results uninterpretable (see Table 2 and Figure 2 for descriptive statistics). This may have resulted due to the aggregation of several ERP components (i.e., P3a, MMN, and Visual P100) and disparate cognitive performance tasks (i.e., VTT, PVT, CCT, and HAT), which yielded significant variability in relative change scores, contributing to the models’ inability to converge. Independent of any environmental stressors, the literature on sex differences in electrophysiology are mixed, with some studies showing sex differences in ERP amplitude (e.g., Bourisly and Shuaib, 2018) and some showing no effect of sex (e.g., Sangal & Sangal, 1996). Moreover, the literature on sex differences in cognitive function and ability is even murkier due to the inability to control the confounding influence of environmental factors like culture, parenting, and learned behavior. While some evidence of anatomical or functional differences exists, none of these observations demonstrate consistent and meaningful sex differences in cognitive or related brain functions (Purves, 2012). Together, these results suggest that a prospective study using the same neural and cognitive measures during acute hypoxia is needed to determine if there are sex differences in these functional measures.

The current study is among the first to examine sex differences within aerospace medicine research to better support all aviators’ operational health and safety. Strengths include a sizeable male-female participant sample and self-reported and objective physiological data collection to validate the hypoxia exposure responses. Yet, it is not without its limitations. First, participants in these studies were not exclusively military aviators; thus, the findings lack some generalizability to the tactical aviation community. The male-female ratio was unequal, and we encourage studies to recruit balanced male-female participant samples, including sex as a covariate in future works. Finally, retrospective data analyses do not establish cause-and-effect relationships yet provide fruitful information concerning the predictor’s degree of explained variances, founding a strong basis for future experimental research.

In conclusion, we found significantly lower SpO2 and increased report of headache for females compared to males during acute hypoxia exposure. These findings add to the small but recently growing area of research into potential sex differences in response to hypoxic challenge. The study’s results bridge sex-specific disparities in aerospace medicine and operational health to refine hypoxia familiarization training within aviation safety programs, enhance the precision of pilot monitoring sensor development, and update emergency response and recovery protocols in the event of a hypoxia occurrence suitable for all aircrew. We recommend continuing to elucidate the impact of sex and intrapersonal differences in hypoxia and other aeromedically relevant stressors on physiological, sensory, and cognitive performance.

## Copyright statement

Some authors are military service members or federal employees of the U.S government. This work was prepared as part of their official duties. Title 17 U.S.C. §105 provides that copyright protection under this title is not available for any work of the U.S. Government. Title 17 U.S.C. §101 defines a U.S. Government work as a work prepared by a military Service member or employee of the U.S. Government as part of that person’s official duties.

## Data Availability

The original contributions presented in the study are included in the article/Supplementary Materials, further inquiries can be directed to the corresponding author.
